# Early esophageal adenocarcinoma detection using deep learning methods

**DOI:** 10.1007/s11548-019-01914-4

**Published:** 2019-01-22

**Authors:** Noha Ghatwary, Massoud Zolgharni, Xujiong Ye

**Affiliations:** 1 University of Lincoln, Lincoln, UK; 2 Arab Academy for Science and Technology, Alexandria, Egypt

**Keywords:** Deep learning, Esophageal adenocarcinoma detection, Barrett’s esophagus, HD-WLE

## Abstract

**Purpose:**

This study aims to adapt and evaluate the performance of different state-of-the-art deep learning object detection methods to automatically identify esophageal adenocarcinoma (EAC) regions from high-definition white light endoscopy (HD-WLE) images.

**Method:**

Several state-of-the-art object detection methods using Convolutional Neural Networks (CNNs) were adapted to automatically detect abnormal regions in the esophagus HD-WLE images, utilizing VGG’16 as the backbone architecture for feature extraction. Those methods are Regional-based Convolutional Neural Network (R-CNN), Fast R-CNN, Faster R-CNN and Single-Shot Multibox Detector (SSD). For the evaluation of the different methods, 100 images from 39 patients that have been manually annotated by five experienced clinicians as ground truth have been tested.

**Results:**

Experimental results illustrate that the SSD and Faster R-CNN networks show promising results, and the SSD outperforms other methods achieving a sensitivity of 0.96, specificity of 0.92 and *F*-measure of 0.94. Additionally, the Average Recall Rate of the Faster R-CNN in locating the EAC region accurately is 0.83.

**Conclusion:**

In this paper, recent deep learning object detection methods are adapted to detect esophageal abnormalities automatically. The evaluation of the methods proved its ability to locate abnormal regions in the esophagus from endoscopic images. The automatic detection is a crucial step that may help early detection and treatment of EAC and also can improve automatic tumor segmentation to monitor its growth and treatment outcome.

## Introduction

A major health problem that has been emerging is esophageal adenocarcinoma (EAC) which is considered the early stage of esophageal cancer. Studies show that esophageal cancer patients hold a 5-year survival rate of only 18.8% [1]. The primary premalignant cause of reaching esophageal malignancy is Barrett’s esophagus (BE) [2, 3], where the development of healthy cells in the esophagus lining into columnar mucosa through metaplastic change leads to EAC [4]. The early detection and treatment of EAC may help in increasing the survival chance of the patient [5].

The process of detection is done through endoscopic examination, high-definition white light endoscopy (HD-WLE) is the primary tool used [6], and the cell deformation stages are confirmed by taking biopsy samples from the surface of the esophagus lining [7]. The appearance and properties of the BE or EAC have challenges in the detection process as it can be located randomly throughout the esophagus tube [8]. Also, the accurate detection requires a physician with significant experience and they are often overlooked during endoscopy surveillance [9]. In addition to that, patients are required to have regular follow-ups through endoscopy examination to control the development of abnormalities into later stages. With the increase in the number of patients, computer-aided detection (CAD) systems have grabbed attention more frequently. There exists an amount of research available in the literature for automatic detection, segmentation and classification that employs several endoscopies such as *white light endoscopy* (WLE), *narrow band imaging* (NBI), *volumetric laser endomicroscopy* (VLE), *confocal laser endomicroscopy* (CLE) and *chromoendoscopy*; these methods are summarized and discussed in [10, 11]. In the next section, an overview of the previous studies on EAC detection from HD-WLE will be discussed.

Recently, deep learning (DL) has been tremendously useful in a wide range of different applications, such as computer vision, natural language processing, medical imaging analysis and much more [12]. Deep learning, specifically, Convolutional Neural Networks (CNNs), has become a conventional technique in medical image analysis (detection, classification, segmentation, etc.) [13]. In this work, we take advantage of recent development in object detection methods that utilize CNNs to locate EAC abnormalities in esophagus endoscopic images by employing the state-of-the-art CNN methods and evaluating them on our dataset. To the best of our knowledge, no work has been addressed before to comprehensively assess the performance of different CNN-based detection methods for detecting tumors in esophageal endoscopic images.

The rest of the paper is organized as follows: the second section represents the related work of EAC detection from HD-WLE images. In the third section the materials and methods are discussed, where a brief description of state-of-the-art deep learning object detection methods is presented, and the dataset used is described, while the experimental results are demonstrated in the fourth section. Finally, the evaluated results are discussed in the fifth section and concluded in the sixth section.

## Related work

Different studies have been conducted in the literature that focused on the detection of BE and EAC using several endoscopic tools. These methods are discussed in [10, 11]. In this section, we will only discuss previous methods that address the detection of EAC abnormalities using the same HD-WLE images dataset that we used in our evaluation.

An evaluation of different texture features extracted from HD-WLE Barrett’s esophagus images was proposed by Sestio et al. [14] and Sommen [15]. This study extracted the following features: texture spectrum, histogram of oriented gradients (HOG), local binary pattern (LBP), Gray Level Co-occurrence Matrix (GLCM), fourier feature, dominant neighbor structure (DNS) and gabor features to compare between them on the effect of EAC detection. As a preprocessing phase, the irrelevant textures tiles have been discarded before applying the classifier. Additionally, the principal component analysis (PCA) was used for reducing the features dimension, and they were classified using the *support vector machine* (SVM). After testing different combinations, this comparison concluded that the merge between gabor and color features achieved the best results compared to other combination of extracted features achieving an overall accuracy of 96.48%. Based on the conclusion in [14, 15], Sommen et al. [9] proposed a CAD system to detect and annotate EAC regions in HD-WLE. Using a Leave-One-Patient-Out Cross-Validation (LOPO-CV) approach the experiments had an 85.7% accuracy compared to the annotation of the specialist with a recall of 0.95 and precision of 0.75 using the SVM classifier on the extracted gabor and color features. More tests were conducted in [16] with the same model on a more substantial dataset that resulted in a sensitivity of 0.86 and a specificity of 0.87 when using SVM and 0.90 and 0.75 for the precision when classified using the Random forest in [17].

Souza Jr. et al. [18] proposed an investigation of the feasibility of the SVM to classify lesions in Barrett’s esophagus based on Speed-Up Robust Features (SURF) descriptors. Two experiments were carried out by extracting the SURF features from the full image and another from the EAC ground truth regions annotated by experts. The results based on full images analysis showed a sensitivity of 0.77 and specificity of 0.82, while the abnormal region-based approach has a sensitivity of 0.89 and specificity of 0.95. These results were analyzed based on the LOPO-CV approach and SVM classifier. Later on, Souza Jr. et al. [19] proposed an Optimum-Path Forest (OPF) classifier to identify BE and adenocarcinoma HD-WLE images. Features were extracted from the images using the Scale-Invariant Feature Transform (SIFT) and the SURF to design a bag of visual words (BoW) to be an input for the OPF and SVM classifiers. Results showed that the OPF outperformed the SVM with sensitivity of 73.2% (SURF)–73.5% (SIFT), specificity of 78.2% (SURF)–80.6% (SIFT) and accuracy of 73.8% (SURF)–73.2% (SIFT).

Mendel et al. [20] studied the analysis of BE using CNN to classify patches in an HD-WLE image into cancerous and non-cancerous. Regarding the experiments, the image was first divided into non-overlapping $224 \times 224$ patches and sampled as cancerous and non-cancerous based on a certain threshold *t*. Each patch has an output probability that was compared to the value *t* to decide whether it is a cancerous region or not. The deep residual network (ResNet) [21] was used as the deep learning method for feature extraction and classification from each patch. After testing the performance of classification at seven different values for threshold *t*, the best performance was achieved at *t* = 0.8 resulting in a sensitivity of 0.94, specificity of 0.88 and *F*-measure of 0.91.

**Fig. 1:**
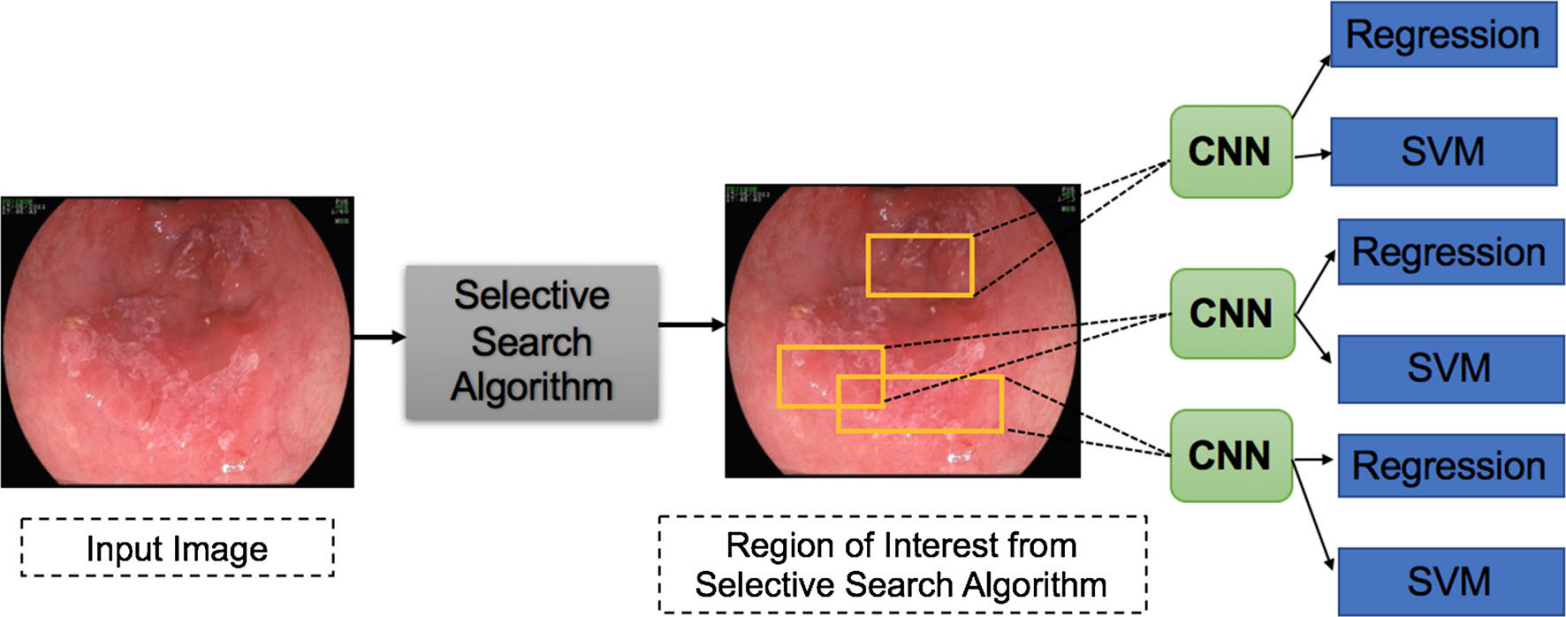
General architecture of the R-CNN. The selective search algorithm is firstly applied to find abnormal candidate regions. The SVM is then used to classify the class based on the feature map from the CNN applied to candidate regions, and the linear regression is used to adjust the bounding box location

## Materials and methods

Traditional object detection methods usually rely on handcrafted features by studying the performance of extracting different features and applying a proposed classification/search method [22]. Deep learning, especially CNNs, has proved its efficiency in various fields such as detection, classification and segmentation [13, 23–25]. There exist various state-of-the-art object detection methods that use deep learning. In this paper, we adopt the following methods Regional-based Convolutional Neural Network (R-CNN), Fast R-CNN, Faster R-CNN and Single-Shot Multibox Detector (SSD) to detect EAC abnormalities. Each of these methods is explained briefly in the following subsection. Additionally, the dataset utilized in the current evaluation is described in details.

### Object detection CNN-based methods

#### Regional-based Convolutional Neural Network (R-CNN):

Girshick et al. [26] first proposed a Regional-based Convolutional Neural Network (R-CNN) as a leading framework for general object detection method using deep learning. The R-CNN method is composed of three main steps as shown in Fig. 1. First, the input image is scanned to generate over 2000 region proposals that might contain objects based on a selective search algorithm [27]. The goal of the selective search algorithm is to provide several candidate regions that belong to an object. It starts by generating an initial sub-segmentation to find a small set of independent class objects. Then it keeps repeating combining the similar regions into larger ones using the greedy algorithm to find the most similar ones. Finally, it outputs candidate regions called proposals that contain objects. After that, a CNN is run over each of the proposal to extract features from this region. Finally, the extracted features from the previous step are fed into an SVM classifier to classify this region into a suspected object and a Linear regressor is used to refine the bounding box if the object exists. The method merged between the original region proposal methods with CNNs, but it was considered slow for real-time processing and computationally expensive in the training process.

#### Fast R-CNN

**Fig. 2:**
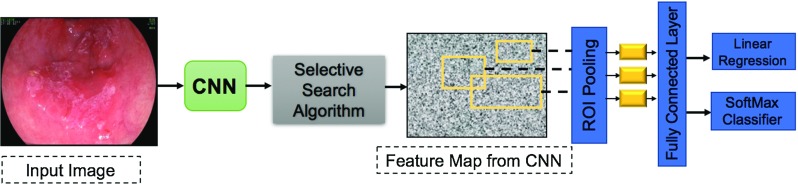
General architecture of the Fast R-CNN. The CNN is applied to the input image to extract the feature map, and the selective search algorithm is performed to find abnormal candidate regions. The ROI is applied after that to unify the feature vector size for classification using Softmax classifier

To overcome the R-CNN drawbacks, Girshick proposed the Fast R-CNN [28] through two main modifications. Firstly, the CNN feature extraction is performed over the image itself rather than over the proposed regions. Therefore, the generated region proposals are based on the last feature map from the network, and the CNN is only trained once on the full image. Secondly, the SVM classifier is replaced with a single softmax layer that outputs a class probability instead of running multiple SVMs for various object classes. Additionally, an ROI pooling layer is added to the last convolutional layer to unify the feature vector size before applying the softmax classification. The performance of the Fast R-CNN was improved regarding the speed compared to the R-CNN, but the executed selective search algorithm still caused a considerable overhead. The architecture of the Fast R-CNN is illustrated in Fig. 2.

**Fig. 3:**
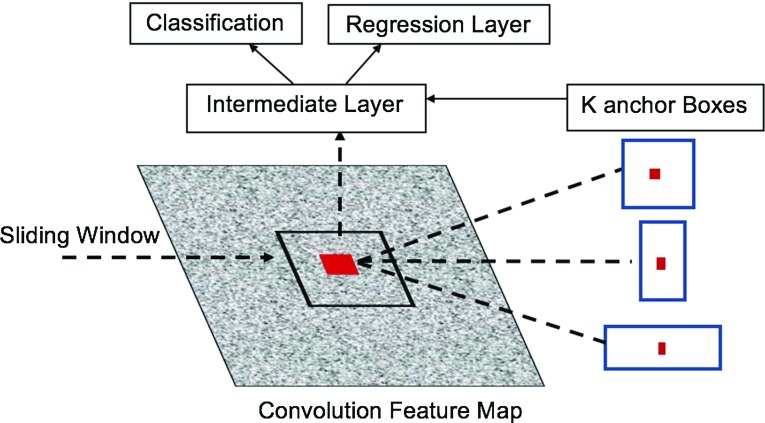
An example of different anchor boxes with different sizes and ratios for a specific location in the RPN stage

#### Faster R-CNN

**Fig. 4:**
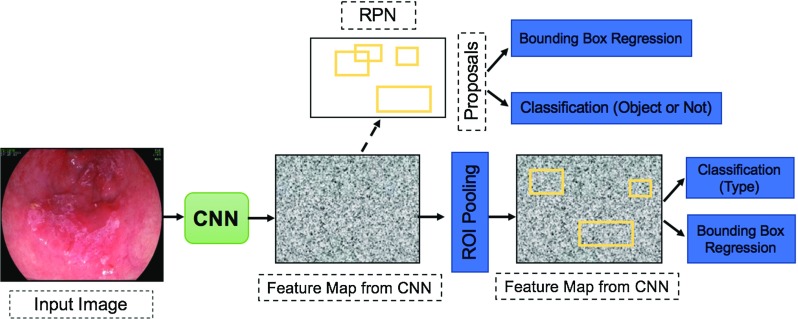
General architecture of the Faster R-CNN. The CNN is applied to the input image to extract the feature map that is later used by both the RPN and the ROI pooling layers (Feature map is shared between both). The RPN outputs the classification score and bounding box location of the candidate region proposals that are passed on to the next stage. The ROI layer unifies the feature vector size of the candidate region proposal that is classified using softmax

Ren et al. [29] suggested combining a proposed Region Proposal Network (RPN) instead of the selective search into the Fast R-CNN leading to a more real-time method called Faster R-CNN. The proposed RPN generates region proposals for each location using the last feature map produced from the CNN based on *anchor boxes*. The anchor boxes are detections boxes that have different sizes and ratios that are compared to the ground truth during the training process. For each location in the feature map, there are *K* different anchor boxes centered around it as shown in Fig. 3. The total number of anchor boxes per image is $(K\times W\times H)$ where the *W* and *H* are the sizes of the last feature map. During training, each generated anchor box is compared to the ground truth object location. Boxes that overlap the ground truth with an Intersection over Union (IoU) based on a certain threshold are considered as an object (no class specified). The IoU is calculated as follows:

1 $$\begin{aligned} \hbox {IoU}= \frac{A_\mathrm{gt} \cap A_\mathrm{p}}{A_\mathrm{gt} \cup A_\mathrm{p}} \end{aligned}$$

where $A_\mathrm{gt}$ is the area of the ground truth bounding box while $A_\mathrm{p}$ is the predicted bounding box from the regression layer. The selected anchor boxes are passed on as region proposals from RPN stage with a classification score for each box and four coordinates that represent the location of this object. Some region proposals highly overlap each other; therefore, non-maximum suppression (NMS) is used to prune the redundant regions leading to a reduced number of region proposals. Later on, the selected region proposals are fed into the next phase as in Fast R-CNN. The ROI pooling divides the input feature map from candidate anchor boxes into a fixed number of almost equal regions. Maxpooling is applied to these regions; consequently, the output from the phase is always fixed size regardless of the input size. One of the main benefits of the Faster R-CNN is that the convolutional layer between two networks (RPN and Fast R-CNN) is shared as shown in Fig. 4 rather than learning two separate networks.

**Fig. 5:**
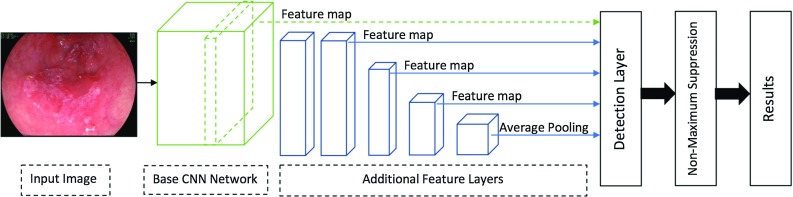
General architecture of the SSD [30]. The SSD is a single unified network for both testing and inference

#### Single-Shot Multibox Detector (SSD)

Liu et al. [30] presented a Single-Shot Multibox Detector (SSD). SSD is considered a faster deep learning object detection method compared to previously discussed methods as it generates the predicting bounding box and classifies the object within it in a single operation while processing the image. During the training process, the SSD takes the image and the ground truth as inputs. Following that, the image is passed through a series of convolutional layers that are combined throughout the network as shown in Fig. 5. The SSD generates a list of bounding boxes for each location using priors (i.e., same as anchors in Faster R-CNN) and then adjusts it to be close to the ground truth location as much as possible. Although the number of generated boxes from SSD is considered a huge number compared to the other methods, it does not guarantee to have an object inside it. An NMS is applied to minimize the number of boxes by grouping the highly overlapping regions and choosing the box with the highest confidence.

Additionally, negative samples are kept with a ratio of 3:1 compared to positive samples in order to apply *hard-negative mining*. The hard-negative mining helps the network to better learn the incorrect detection leading to more accurate results. The backbone CNN network used in the Faster R-CNN and the SSD is the VGG’16 [31] after discarding the fully connected layer and using its feature map. One of the main reasons for using the VGG’16 is that it has a very high performance toward image classification problems.

In this paper, we evaluate the performance of the described deep learning object detection methods using the VGG’16 as the backbone network to identify the EAC abnormalities in the HD-WLE images automatically.

**Fig. 6:**
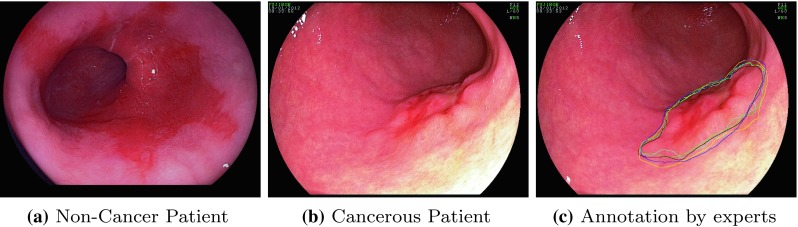
Examples of the HD-WLE images from the provided dataset showing **a** non-cancerous Barrett’s patient, **b** EAC patient and **c** annotation from five different experts

### Dataset

A dataset composed of 100 HD-WLE images of lower esophagus provided by the *Endoscopic Vision Challenge MICCAI 2015* [32] and [9] is used in the evaluation. The 100 images were divided into 50 images with non-cancerous regions (Fig. 6a) and another 50 with EAC (Fig. 6b). The images were gathered from 39 patients, among those patients, 22 patients diagnosed with esophageal adenocarcinoma and 17 patients with non-cancerous Barrett’s. Different numbers of images were captured from each patient resulting in a varied number from one to eight images per patient. Lesions found in the abnormal images have been annotated by five leading experts in the field to obtain golden standards as shown in Fig. 6c. Due to the differences in manual segmentation from one expert to another, we used the largest intersection area between the annotations from all the experts during the training and testing phase.

## Experiments

In this section, we first give details about the implementation setup for the CNN methods. Then, the measures used in the evaluation process are described. Finally, we evaluate the performance of the detection methods on our dataset.

### Experimental setup

Due to the limited publicly available dataset, we performed an addition data augmentation to the training data by flipping along the axial plane and rotation in different angles with 90$^{\circ }$, 180$^{\circ }$ and 270$^{\circ }$.

For implementation, we adopt the Keras library [33] based on Python to train and test the different deep learning object detection models on a single Nvidia 1080Ti GPU. The VGG’16 was employed as the backbone CNN network for the four discussed models, which has been trained from scratch on the dataset after augmentation. Each model was trained for 5000 iterations with the learning rate set to 0.0001. Additionally, the images were used with its original size ($1600 \times 1200$) for the following networks R-CNN, Fast R-CNN and Faster R-CNN, while in the SSD, the images were rescaled to $300 \times 300$.

During the training process, the anchor boxes sizes and ratios for the RPN stage in the Faster R-CNN were set to the default setting as proposed in [29], where there exist *K* = 9 anchors at each location with three scales ($128^2$, $256^2$ and $512^2$ pixels) and three aspect ratios (1:1, 1:2 and 2:1). Furthermore, the anchor boxes are compared with the ground truth to generate the RPN proposals, and the region with an IoU (Eq. 1) greater than 0.7 is considered as a proposal. On the other hand, the SSD uses multiple feature maps to predict the target location and calculate a confidence score. In the evaluation, the features are extracted at convolutional layers 4 and 7. Also, the NMS was set to 0.7 for bounding box selection.

### Evaluation measures

To assess the performance of the CNN object detection methods in detecting the tumor regions, we employ the Average Recall Rate (ARR) and Average Precision Rate (APR) [34], to measure the accuracy of the detected bounding box in comparison to the ground truth region in the cancerous images. Also, sensitivity (SE), specificity (SP) and the *F*-measure (FM) are measured over all the test images (non-cancerous and cancerous) as follows:

2 $$\begin{aligned}&\hbox {ARR}=\frac{1}{N} \sum _{I=1}^N \frac{B_{I}^\mathrm{g}\cap B_{I}^\mathrm{p}}{B_{I}^{rm g}} \end{aligned}$$

3 $$\begin{aligned}&\hbox {APR}=\frac{1}{N} \sum _{I=1}^N \frac{B_{I}^\mathrm{g}\cap B_{I}^\mathrm{p}}{B_{I}^\mathrm{p}} \end{aligned}$$

4 $$\begin{aligned}&\hbox {SE}= \frac{\mathrm{TP}}{\mathrm{TP}+\mathrm{FN}} \end{aligned}$$

5 $$\begin{aligned}&\hbox {SP}=\frac{\mathrm{TN}}{\mathrm{TN}+\mathrm{FP}} \end{aligned}$$

6 $$\begin{aligned}&\hbox {FM}= \frac{2 \cdot \mathrm{TP}}{2 \cdot \mathrm{TP}+\mathrm{FP}+\mathrm{FN}} \end{aligned}$$

where *N* is the total number of images, the $B^\mathrm{g}$ is the ground truth bounding box area of the tumor region while $B^\mathrm{p}$ is the area of predicted bounding box proposed by the detection method. Taking into consideration the (*x*, *y*) coordinates as the location of the upper left corner of both boxes to compute the intersection, all measures have been assessed in reference to the cancerous patients, True Positive (TP) the number of cancerous images that had correct prediction, True Negative (TN) the number of non-cancerous images that had correct prediction, False Negative (FN) number of cancerous images that had no prediction and False Positive (FP) number of non-cancerous images that had regions predicted as cancerous.

### Results

The four deep learning object detection approaches discussed in “Object detection CNN based methods” section have been carried on the available dataset after augmentation. The five measures defined in Eqs. 2–6 were used to evaluate detection performances. First, the ARR and APR were used to evaluate the bounding box accuracy. A higher APR demonstrates that a more significant region is overlapping between the predicted region and the ground truth, and a higher ARR shows that the tumor region generated by the detection method excludes more non-cancerous areas. Moreover, the sensitivity, specificity and *F*-measure rates were measured, where the number of the missed region in a cancerous patient (no detection) and any false prediction in normal patient images affected the results. Additionally, if the IoU value between the generated bounding box and the ground truth is less than 0.5, then the produced bounding box is considered to be a false prediction (non-cancerous). Furthermore, the time for the detection processes for each method was measured in seconds during the testing phase.

The experiments have been carried out using three types of validation. *Experiment 1*: from the 39 patients, 60% were used for training [21 patients (*12 cancerous, 9 non-cancerous Barrett’s*)], 20% for validation [9 patients (*5 cancerous, 4 non-cancerous Barrett’s*)] and 20% for testing [9 patients *(5 cancerous, 4 non-cancerous Barrett’s)*]. The experiments were carried twice to verify the results using more cases by changing the patients dataset between the validation and testing sets in the second experiment. Therefore, the results presented in Table 1 are based on a total of 18 patients (10 cancerous and 8 non-cancerous Barrett’s) that are entirely different from the dataset used for training the model. *Experiment 2*: The dataset was evaluated based on 5-fold-cross-validation (5-fold-CV), where the dataset is divided into 5-fold randomly. (Each fold will hold 7–8 patients.) The results of the second experiment are shown in Table 2. *Experiment 3*: Leave-One-Patient-Out cross-validation (LOPO-CV) is applied to compare the four detection methods. Table 3 demonstrates the results from LOPO-CV experiment in addition to a comparison with two of state-of-the-art (Mendel et al. [20] and Sommen et al. [16]) methods that use the same dataset. The results of the three experiments will be discussed further in the following section.

**Table 1:** Average Recall Rate (ARR), Average Precision Rate (APR), sensitivity (SE) and specificity (SP) and *F*-measure (FM) for the state-of-the-art object detection deep learning methods on the EAC dataset based on 60% training and 40% testing

Method	APR	ARR	SE	SP	FM	Time (s)
R-CNN	0.43	0.41	0.47	0.41	0.44	13.38–37.81
Fast R-CNN	0.66	0.37	0.53	0.57	0.55	0.65–2.1
Faster R-CNN	0.50	0.78	0.72	0.83	0.83	0.3–0.45
SSD	**0.69**	**0.81**	**0.93**	**0.93**	**0.93**	0.1–0.2

Bold values represent the highest values

**Table 2:** Average Recall Rate (ARR), Average Precision Rate (APR), sensitivity (SE) and specificity (SP) and *F*-measure (FM) for the state-of-the-art object detection deep learning methods on the EAC dataset based on 5-fold-CV

Method	APR	ARR	SE	SP	FM
R-CNN	0.48	0.41	0.50	0.40	0.48
Fast R-CNN	0.62	0.43	0.64	0.64	0.64
Faster R-CNN	0.68	**0.83**	0.78	0.80	0.79
SSD	**0.70**	0.79	**0.90**	**0.88**	**0.88**

Bold values represent the highest values

**Fig. 7:**
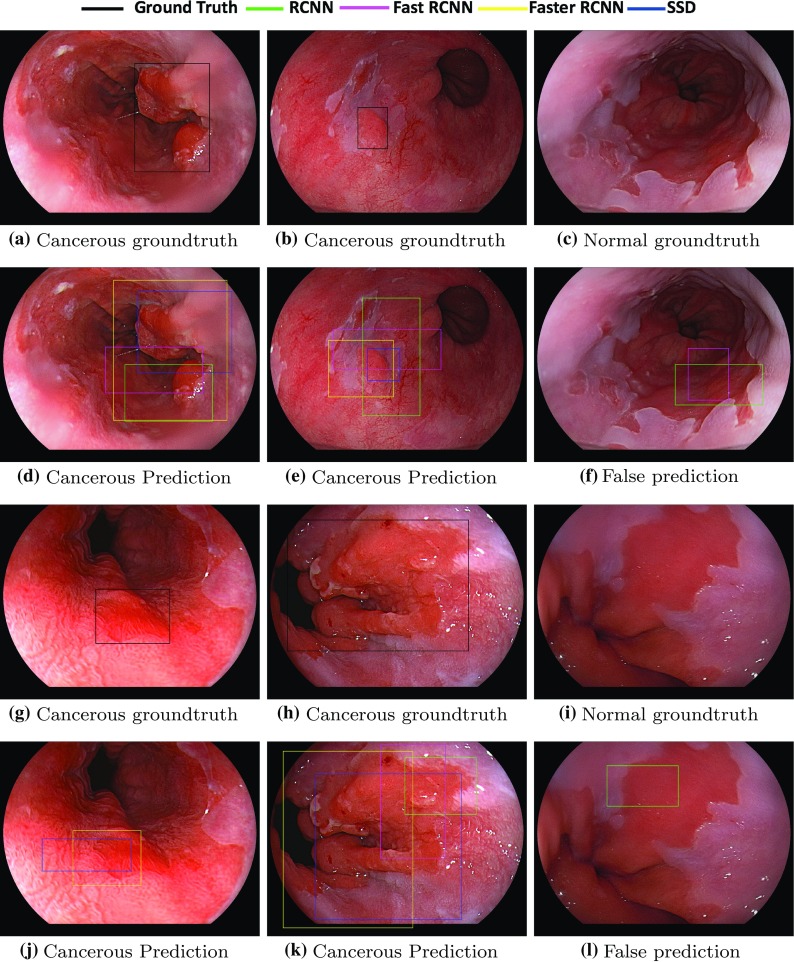
Bounding box ground truth based on experts annotation and the output from the R-CNN, Fast R-CNN, Faster R-CNN and SSD when using 5-fold-CV from different patients showing correct prediction in **d**, **e**, **j** and **k** with different scores and a false prediction on a non-cancerous patient in **f ** and **l**

Furthermore, the bounding box results from each method have been provided on some sample images shown in Fig. 7 and compared to the ground truth bounding box. The figure shows different samples of the true and false positives detection. An example from one non-cancerous image that had false prediction by the R-CNN and Fast R-CNN method is shown in Fig. 7c, and another one by the R-CNN is shown in Fig. 7l. Moreover, Fig. 7j illustrates the detection of Faster R-CNN and SSD only as the other two methods failed to find an EAC region. The rest of the figures demonstrate the performance of the four models in detecting the abnormal regions in minor and complex tumors.

**Table 3:** Average Recall Rate (ARR), Average Precision Rate (APR), sensitivity (SE), specificity (SP) and *F*-measure (FM) for the state-of-the-art object detection deep learning methods on the EAC dataset based on LOPO-CV

Method	SE	SP	FM
R-CNN	0.60	0.56	0.59
Fast R-CNN	0.64	0.60	0.63
Faster R-CNN	0.88	0.86	0.87
SSD	**0.96**	**0.92**	**0.94**
Mendel et al. [20]	0.94	0.88	0.91
Sommen et al. [16]	0.86	0.87	0.87

Bold values represent the highest values

## Discussion

CAD has been acting as an essential tool in clinical practice and research by providing a second opinion to the clinician. With the evolving of the use of deep learning methods in implementing CAD methods in various fields, there has been a tremendous improvement in accuracy. Multiple CAD systems have been proposed in the literature that mainly relied on handcrafted features to detect EAC abnormalities in endoscopic images. Only one method used the deep learning to classify the patches inside image into cancerous and non-cancerous [20].

The APR and ARR are used to measure the performance of the detection methods by evaluating the output bounding box in cancerous images only. They both measure the overlapping region between the predicted bounding box and ground truth. As shown in Table 1, the APR results for the Fast R-CNN and the SSD achieved 0.66 and 0.69, respectively. Additionally, the APR results from Table 2 show that the Faster R-CNN achieved 0.68, while the SSD achieved 0.70. From both tables, the SSD proved the ability to detect a greater abnormal region that overlapped with the ground truth generated by experts compared to the other three CNN methods. Moreover, the ARR from these two tables, the Faster R-CNN and SSD outperform the Fast R-CNN and R-CNN with results of 0.78 and 0.81 from Table 1 and 0.83 and 0.79 from Table 2. The results indicate that the SSD and Faster R-CNN were able to detect fewer false positive regions (non-cancerous areas) inside the generated bounding box for the abnormal area.

Additionally, the sensitivity, specificity and *F*-measure are measured for the three experimental validation methods. Results in Table 1 are based only on 18 patients (10 cancerous and 8 non-cancerous Barrett’s) as described previously in “Results” section. The SSD outperforms among the compared methods with a result of 0.93 for the three measures. The high sensitivity of the SSD result from this table indicates that it had a good performance in detecting EAC regions from the cancerous images and less false bounding boxes in the non-cancerous Barrett’s images. The Faster R-CNN followed by with results of 0.72 for the sensitivity and 0.83 for both the specificity and *F*-measure.

From Table 2 based on 5-fold-CV. The SSD surpasses the other three methods with a sensitivity of 0.90, both specificity and *F*-measure of 0.88. The results demonstrate that the SSD had a high performance in generating bounding boxes that located in abnormal regions throughout the testing dataset and less false ones. For the Faster R-CNN as shown in Table 2, the results of the sensitivity were 0.78 and 0.80 for the specificity and 0.79 for the *F*-measure demonstrating an acceptable performance.

As a further study, a comparison of the results with other state-of-the-art models provided by Mendel et al. [20] and Sommen et al. [16] is illustrated in Table 3. For a fair evaluation, we employ the same validation method LOPO-CV. Firstly, the sensitivity was evaluated, and the SSD achieved the highest performance among the four deep learning methods and surpassed the results of [20] by 2% and [16] by 10%. Also, the Faster R-CNN outperformed against [16] by 2%. Additionally, the specificity of the SSD achieved 92% indicating the improvement of less false positives regions, while the Faster R-CNN achieved 0.86 that is considered comparable with results of [20] and [16].

As observed in Tables 2 and 3, the R-CNN and the Fast R-CNN have the lowest performance. The reason behind this is that both methods rely on selective search algorithm to generate a region of interest. As explained in the earlier section, selective search algorithm uses the greedy algorithm to search for a location for object localization. The greedy algorithm has limitations in finding the optimal solution. Additionally, the grouping process is done based on the color space difference and similarity metrics, while for our dataset, it is difficult to differentiate between non-cancerous Barrett’s regions and EAC solely based on color as they both have a darker color than normal regions which might lead to more false positives. On the other hand, the use of anchor boxes and priors in the Faster R-CNN and the SSD helps improve the performance of generating more candidate regions of interest. Furthermore, the results of Table 3, in general, are more improved than that in Table 2 as the LOPO-CV allows more dataset to be trained than the 5-fold-CV.

The differences in sensitivity and specificity between the four object detection methods were statistically evaluated using the paired *t*-test at a confidence level of 95%. The results of the two-tailed *p* value of the two best performers (SSD and Faster R-CNN), when compared with the other two methods, are illustrated in Table 4. As shown, the difference between the sensitivity and specificity of the SSD and Faster R-CNN was found to be significantly different when they were compared to the R-CNN and Fast R-CNN using the *t*-test. Additionally, the *t*-test was also employed to determine whether there are any statistical differences in the sensitivity and specificity, obtained using the two validation methods (i.e., 5-fold-CV and LOPO-CV). The *p* value of the sensitivity and specificity for each deep learning object detection method was as follows R-CNN (*0.0235,0.0068*), Fast R-CNN (*0.3222, 0.1594*), Faster R-CNN (*0.0238 ,0.0832*) and SSD (*0.0832, 0.1594*). Our analysis based on these *p* values suggests that the two validations for the R-CNN and Faster R-CNN show a significant difference. On the other hand, the difference in results for the SSD and the fast R-CNN is not statistically significant.

**Table 4:** The *p*-value calculate using the *paired t*-test to measure the difference of sensitivity and specificity results between the four deep learning methods

Method	Sensitivity		Specificity	
	R-CNN	Fast R-CNN	R-CNN	Fast R-CNN
Faster R-CNN	0.0049	0.1279	0.0001	0.0443
SSD	0.0012	0.0882	0.0001	0.0036

Moreover, the detection time during testing was measured in seconds for each method as shown in Table 1. The time started with a range of 13.38–37.81 s when using the R-CNN and then decreased while using a more updated method. The R-CNN requires a significant amount of time as it generates around 2000 region proposal for each location and then used to extract features from them using CNN. This leads to a repetition of almost 2000 times to extract features from one image. The detection time drops to 0.65–2.1 s when using the Fast R-CNN, as the selective search is applied to the extracted features after applying the CNN to the input image. The Faster R-CNN was faster after sharing the weights and feature map between the RPN and ROI pooling layer resulting in a range of 0.3–0.5 s to generate detection bounding boxes. The SSD surpassed against the other methods in predicting region in most of the cancerous images with only 0.1–0.2 s. The reason for this is that the SSD can localize the object and classify it in a single forward pass network. We believe that with a more powerful hardware (i.e., Nvidia Titan, Nvidia Tesla V100), the detection speed would be further increased.

In addition to providing the quantitative evaluation, we also randomly choose some qualitative results of the deep learning object detection methods for different cases as shown in Fig. 7. For example, Fig. 7e demonstrates that the different methods can detect some difficult instances in which the abnormality is located in a small region and is visually similar to other areas inside the same image. Also, in Fig. 7d, k the abnormal areas are present in most of the images. The SSD and Faster R-CNN show the ability to detect most of the EAC area compared to the ground truth. Furthermore, Fig. 7f, l lists some false positive regions detected by the R-CNN and Fast R-CNN. The non-cancerous Barrett’s from normal patients have a difference in color in some areas as shown in Fig. 7c, i which makes the detection challenging. The accuracy of this bounding box is discussed earlier using the ARR and APR values compared to the ground truth and illustrated in Fig. 7.

The esophagus has a special internal structure that makes it challenging to differentiate between normal and abnormal regions. Also, the abnormalities inside the esophagus are particularly challenging due to its different sizes, locations and shapes. There exist variations in the size and the location in the generated bounding boxes from the four models, where each box might include non-cancerous regions. Table 5 calculates the average error presented by each model in capturing non-cancerous regions inside the bounding box. As shown, the R-CNN and Fast R-CNN presented higher error percentage compared to the other two models. This indicates the bounding box generated by these two methods included a high ratio of non-cancerous regions. On the other hand, the Faster R-CNN and SSD provided a lower error rate for containing non-cancerous areas; therefore, they were able to provide better bounding boxes localized around the cancerous regions.

**Table 5:** Average error presented by each model in capturing non-cancerous regions inside the produced bounding boxes in the EAC images

	R-CNN	Fast R-CNN	Faster R-CNN	SSD
Average error	0.388	0.328	0.211	0.197

## Conclusion

In this paper, we adapted the state-of-the-art deep learning object detection methods to automatically identify the EAC abnormalities from HD-WLE images. Throughout the evaluation experiments, the SSD has proved to be the leading performance regarding the different evaluation measures, with an outstanding result of 0.90 for the sensitivity, 0.88 for the specificity and 0.88 for the *F*-measure when evaluated based on 5-fold-CV.

Also, the average precision and recall rates are of 0.70 and 0.79 for the SSD and 0.68 and 0.83 for the Faster R-CNN in locating abnormal regions compared to the expert’s annotation. The current study is a step forward to use deep learning object detection methods to find abnormalities in esophageal endoscopy still image. We mainly focused on detection by using the bounding boxes to allocate abnormal regions. Additionally, experiments based on LOPO-CV have been carried out and compared with other state-of-the-art methods. The SSD and Faster R-CNN were able to surpass among the results.

Moreover, figures have been presented to illustrate the generated bounding box by each method. There are some errors introduced by the bounding boxes by the different models that need to be improved. The CNN network used for feature extraction can be modified/replaced with adjustments in network parameters to improve the final detection performance.

Further work will be held to improve the performance of automatic EAC detection using the most efficient methods in current evaluation ( i.e., SSD and Faster R-CNN) and will include more patients data to assess the proposed modified methods further.
